# Early warning system hypertension thresholds to predict adverse outcomes in pre-eclampsia: A prospective cohort study

**DOI:** 10.1016/j.preghy.2017.11.003

**Published:** 2018-04

**Authors:** Hannah L. Nathan, Paul T. Seed, Natasha L. Hezelgrave, Annemarie De Greeff, Elodie Lawley, John Anthony, David R. Hall, Wilhelm Steyn, Lucy C. Chappell, Andrew H. Shennan

**Affiliations:** a Women’s Health Academic Centre, 10th Floor, North Wing, St Thomas’ Hospital, Westminster Bridge Road, King’s College London, London SE1 7EH, UK; b Maternity Centre, Groote Schuur Hospital, University of Cape Town, Main Road, Observatory, Cape Town 7935, South Africa; c Department of Obstetrics and Gynaecology, Tygerberg Hospital, Stellenbosch University, Francie Van Zijl Drive, Cape Town 7500, South Africa

**Keywords:** Pre-eclampsia, Hypertension, Blood pressure, Early warning system

## Abstract

**Objectives:**

To evaluate the association between blood pressure (BP) measurements and adverse outcomes in women with pre-eclampsia.

**Study design:**

A prospective cohort study of women with pre-eclampsia admitted to three South African tertiary facilities. BP was measured using the CRADLE Vital Signs Alert (VSA), incorporated with a traffic light early warning system; green: systolic BP <140 mmHg and diastolic BP <90 mmHg, yellow: systolic BP 140–159 and/or diastolic BP 90–109 mmHg (but neither is above the upper threshold), red: systolic BP ≥160 mmHg and/or diastolic BP ≥110 mmHg.

**Main outcome measures:**

Maternal: death, eclampsia, stroke, kidney injury; process measures: magnesium sulfate use, Critical Care Unit (CCU) admission; perinatal: stillbirth, neonatal death, preterm delivery.

**Results:**

Of 1547 women with pre-eclampsia (including 42 twin pregnancies), 33.0% of women triggered a red light on admission and 78.6% at their highest BP. Severe hypertension and adverse outcomes were common across yellow and red categories. Comparing admission red to yellow lights, there was a significant increase in kidney injury (OR 1.74, CI 1.31–2.33, trend test p = .003), magnesium sulfate use (OR 3.40, CI 2.24–5.18, p < .001) and CCU admission (OR 1.50, CI 1.18–1.91, p < .001), but not for maternal death, eclampsia, extended perinatal death or preterm delivery.

**Conclusion:**

The CRADLE VSA, with integrated traffic light early warning system, can identify women who are hypertensive, at increased risk of severe pre-eclampsia complications and in need of escalation of care. Women who triggered a red light were at increased risk of kidney injury, magnesium sulfate use and CCU admission.

## Highlights

• The CRADLE Vital Signs Alert is designed to risk stratify women with pre-eclampsia.
• A yellow or red light corresponds to increased risk of pre-eclampsia complications.
• Those who trigger a yellow or red light need escalation of care.

## 1 Introduction

Pre-eclampsia affects 3–5% of pregnancies and is a leading cause of maternal and perinatal mortality and severe morbidity globally [1], [2]. In high-income countries, maternal mortality from pre-eclampsia is now rare; this is a result of prompt action following diagnosis facilitated by blood pressure (BP) and urinary dipstick proteinuria measurement [3]. In low- and middle-income countries (LMIC) settings, where 99% of all maternal deaths occur, healthcare providers often do not have access to the necessary equipment, the training to use the equipment and respond appropriately to abnormal vital signs, nor access to effective referral pathways [4]. It is in LMICs that women are dying from preventable complications of pre-eclampsia.

The CRADLE Vital Signs Alert (VSA) is a hand-held, upper-arm, semi-automated device measuring BP and pulse to facilitate prompt recognition of abnormalities in vital signs. It has been designed specifically for healthcare providers from LMICs and meets the World Health Organisation’s requirements for use in low-resource settings [5]. Device accuracy has been validated for use in pregnancy, including pre-eclampsia and low BP in pregnancy [6], [7], [8]. The device incorporates a traffic light early warning system, aimed at alerting all healthcare providers (regardless of training) to vital sign abnormalities secondary to pre-eclampsia, maternal haemorrhage and sepsis. For pre-eclampsia, well-recognised thresholds for diagnosis have been selected for the thresholds triggering the lights (green = systolic BP <140 mmHg and DBP <90 mmHg, yellow = systolic BP 140–159 and/or diastolic BP 90–109 mmHg (but neither is above the upper threshold), red = systolic BP ≥160 mmHg and/or diastolic BP ≥ 110 mmHg) [9].

Although clinicians rely on these recommended BP thresholds to guide diagnosis and management of pre-eclampsia, the thresholds that indicate increased risk of complications of pre-eclampsia and (therefore dictate management) are based on expert opinion and limited data [9], [10], [11], [12], [13], [14]. This study aimed to determine whether recommended BP thresholds (that trigger yellow and red lights in the CRADLE VSA) are associated with adverse outcomes in women with pre-eclampsia at facility-level in South Africa.

## 2 Methods

This prospective observational cohort study was undertaken between January 2015 and May 2016 at three state tertiary-level maternity units in South Africa (Groote Schuur, Tygerberg and Kimberley Hospitals). Women were eligible if they had a clinical diagnosis of pre-eclampsia during their admission. There were no exclusion criteria.

The study was approved by the Stellenbosch University Ethics Committee (N14/06068), University of Cape Town Ethics Committees (410/2014) and the University of the Free State Ethics Committee (230408-011). Local ethics committees at two of the three sites (Tygerberg Hospital and Kimberley Hospital) required individual informed written consent to be obtained before the woman was enrolled in the study (or waiver of consent was granted if the woman was unconscious). Institutional-level agreement for the study was given at the third site – Groote Schuur Hospital (i.e. individual-level consent was not required).

All BP devices in the three maternity units, except those within the anaesthetic and recovery areas, were replaced by the CRADLE Vital Signs Alert (VSA). Management protocols were unaltered.

BP on admission (‘admission BP’) and the highest BP during the course of the woman’s hospital stay (‘highest BP’) were recorded for each woman. Pre-specified adverse clinical outcomes were recorded and included maternal outcomes (death, eclampsia, stroke, kidney injury), process measures (maternal use of magnesium sulfate, maternal Critical Care Unit (CCU) admission) and perinatal outcomes (extended perinatal death, delivery at <34 weeks and <37 weeks of gestation). Kidney injury was defined as highest creatinine during admission ≥90 μmol/L. Critical Care Unit admission was defined as admission to a critical care area providing at least additional monitoring and interventions [15]. Extended perinatal death included stillbirth, early neonatal and late neonatal death [16]. Data were extracted through patient notes reviewed by a local researcher and independently adjudicated. All women with pre-eclampsia were included but those with missing outcomes were excluded for that particular outcome analysis.

The primary analysis was the relationship between clinical outcomes to the BP thresholds that trigger the CRADLE VSA traffic light early warning system, using non-parametric trend testing [17], odds ratios (with 95% confidence intervals) and post-test probability (with 95% confidence intervals) for outcomes. Post-test probability (defined as the proportion of women triggering each traffic light who have the outcome) and odds ratios for yellow compared to green and red compared to yellow traffic lights were calculated. The post-test probability was reported rather than sensitivity, specificity, and positive and negative predictive values related to a single threshold (as recommended by Sackett et al.) [18] and 95% confidence intervals were included to allow for generalisation from the sample to the population with similar characteristics. The clinical outcomes associated with ‘highest’ SBP was assessed using Area Under the Receiver Operator Characteristic Curve (AUROC). Absolute differences in outcomes at increasing ‘highest’ SBP was illustrated using line graphs. Stepwise logistic regression analysis explored possible inflection points of ‘admission’ and ‘highest’ systolic and diastolic BP across the outcomes, including at the traffic light thresholds of SBP 140 mmHg, SBP 160 mmHg, DBP 90 mmHg and DBP 110 mmHg. For perinatal outcomes, an adjustment for clustering was made, using semi-robust standard errors, to allow for the inclusion of multi-fetal pregnancies.

A post-hoc power calculation for two principal outcomes (eclampsia and extended perinatal death) showed that the rate of eclampsia could be estimated to within 0.9% of the true value with 95% confidence and the rate of extended perinatal death could be estimated to within 1.3% of the true value with 95% confidence, based on incidence in previous literature [19]. Statistical analysis was performed in the statistical package Stata (version 11.2), College Station, TX. The study is reported in accordance with STrengthening the Reporting of OBservational studies in Epidemiology (STROBE) guidelines.

## 3 Results

**Fig. 1:**
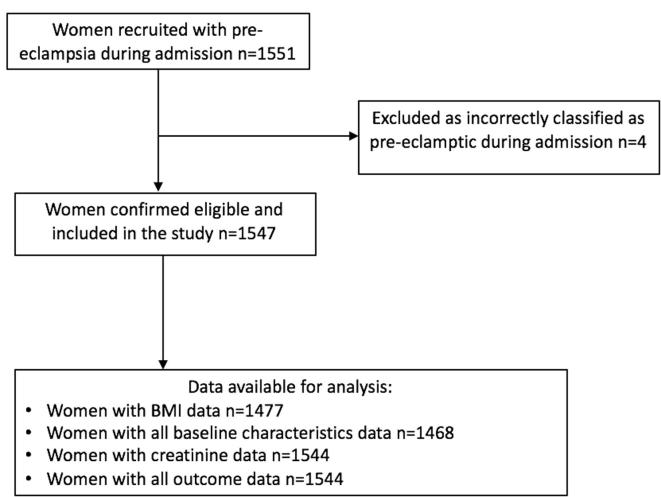
Flow diagram of participants.

A total of 1547 women with pre-eclampsia were eligible, consented and were included in the analysis, with 42 twin pregnancies (Fig. 1). The number of women who declined to take part was not documented. Participant characteristics and BP results are shown in Table 1. 511 (33.0%) women triggered a red light as their ‘admission’ BP and 1216 (78.6%) women triggered a red light at their ‘highest’ BP; nine (0.6%) women did not trigger a yellow or red light as an inpatient i.e. their BP remained within normal limits (their diagnosis of pre-eclampsia was fulfilled by hypertension prior to admission).

**Table 1:** Mean ± standard deviation or number (percentage) of demographic, admission and delivery characteristics.

	All sites	Groote Schuur Hospital	Kimberley Hospital	Tygerberg Hospital
Number of women	1547	770 (49.8)	167 (10.8)	610 (39.4)

*Demographics*				
Age at delivery, year	27.6 ± 6.2	28.0 ± 6.0	28.3 ± 7.2	27.0 ± 6.2
Body mass index, kg/m^2^	30.4 ± 7.73	31.0 ± 8.3	31.9 ± 8.3	29.2 ± 6.7
Multiparous	983 (63.5)	514 (66.8)	117 (70.1)	352 (57.7)

*Admission*				
Gestation on admission, weeks	32.8 ± 4.9	32.0 ± 4.8	33.9 ± 4.7	33.5 ± 4.9
Systolic BP	150 ± 20.6	150 ± 22.3	147 ± 20.0	150 ± 18.2
Diastolic BP	97 ± 15.4	98 ± 15.6	92 ± 16.9	97 ± 14.3

*‘Admission’ light*				
Green	271 (17.5)	136 (17.7)	42 (25.2)	93 (15.3)
Yellow	765 (49.5)	361 (46.9)	85 (50.9)	319 (52.3)
Red	511 (33.0)	273 (35.5)	40 (24.0)	198 (32.5)

*Admission dipstick proteinuria*				
Negative/Trace	165 (10.7)	93 (12.1)	69 (42.6)	3 (0.5)
+1	196 (12.7)	134 (17.4)	15 (9.3)	47 (7.7)
+2	578 (37.5)	245 (31.9)	32 (19.8)	301 (49.3)
+3	601 (39.0)	296 (38.5)	46 (28.4)	259 (42.5)

*Delivery*				
Gestation at delivery, weeks	33.4 ± 4.7	32.8 ± 4.5	34.5 ± 4.4	33.9 ± 4.9
Induction or pre-labour Caesarean section	1357 (87.8)	636 (82.6)	147 (88.6)	574 (94.3)
Caesarean section (pre-labour and emergency)	1060 (69.7)	549 (71.3)	115 (69.3)	417 (68.5)

*‘Highest’ BP (mmHg)*				
Systolic BP	172 ± 16.9	174 ± 17.9	171 ± 16.1	170 ± 15.7
Diastolic BP	104 ± 14.60	106 ± 15.7	102 ± 17.1	103 ± 12.2

*‘Highest’ light during admission*				
Green	9 (0.6)	5 (0.6)	0 (0)	4 (0.7)
Yellow	322 (20.8)	139 (18.1)	42 (25.1)	141 (23.1)
Red	1216 (78.6)	626 (81.3)	125 (74.9)	465 (76.2)

Diastolic BP indicates diastolic blood pressure at the time of ‘highest’ systolic BP; ‘highest’ light during admission indicates the light triggered at the time of ‘highest’ systolic BP.

Table 2 shows the incidence of each outcome. Sixteen (1%) of the women died during their admission. Eclampsia occurred in 147 (9.5%) women and stroke occurred in 4 (0.3%) women. Analysis of characteristics of the four women with stroke was limited; however, mean ‘highest’ SBP was 188 mmHg (SD 33) and mean DBP at the time of ‘highest’ SBP was 114 mmHg (SD 7.7).

**Table 2:** Number (percentage) of maternal, perinatal and process measure outcomes.

	All sites	Groote Schuur Hospital	Kimberley Hospital	Tygerberg Hospital
Number of women	1547	770 (49.8)	167 (10.8)	610 (39.4)

*Maternal outcomes*				
Maternal death	16 (1.0)	3 (0.4)	6 (3.6)	7 (1.1)
Eclampsia (at any time)	147 (9.5)	71 (9.2)	16 (9.6)	60 (9.8)
Stroke (at any time)	4 (0.3)	2 (0.3)	0 (0)	2 (0.3)
Kidney injury	272 (17.6)	174 (22.6)	21 (14.0)	72 (11.8)

*Secondary outcomes*				
*Process measures*				
Maternal magnesium sulfate	1345 (86.9)	686 (89.1)	120 (71.9)	539 (88.4)
Maternal Critical Care Unit admission	453 (29.3)	105 (13.6)	114 (68.3)	234 (38.4)
Total number of infants	**1589**	**793**	**172**	**624**

*Perinatal outcomes*				
Stillbirth	281 (17.7)	162 (20.4)	16 (9.3)	103 (16.5)
Early neonatal death	39 (2.5)	21 (2.6)	6 (3.5)	12 (1.9)
Late neonatal death	12 (0.8)	5 (0.6)	4 (2.3)	3 (0.5)
Preterm birth <34 weeks	544 (41.7)	303 (48.2)	52 (33.3)	189 (36.3)
Preterm birth <37 weeks	913 (70.0)	491 (78.1)	99 (63.5)	323 (62.1)

Table 3, Table 4 show the non-parametric trend test, odds ratios and post-test probability for outcomes for ‘admission BP’ and ‘highest BP’ traffic lights triggered and associated outcomes. Odds ratios for ‘highest BP’ yellow versus green were not calculated as there were too few green lights triggered (n = 9, 0.6% of ‘highest’ lights) for meaningful comparison, as expected in this high-risk cohort.

**Table 3:** Frequency, post-test probability for outcomes (95% CI) of outcomes across green, yellow and red ‘admission’ traffic light thresholds, odds ratios (95% CI) of yellow vs. green and red vs. yellow traffic lights and non-parametric trend test for worsening traffic light triggers (green to yellow to red).

Outcomes	Maternal death	Eclampsia	Kidney injury	Magnesium sulfate use	CCU admission	Extended perinatal death	Delivery <34 weeks	Delivery <37 weeks
*Post-test probability for outcomes (n,%, 95% CI)*								
Green	3/271 1.1 (0.2, 3.2)	26/271 9.6 (6.4, 13.7)	44/271 16.3 (12.1, 21.3)	228/271 84.1 (79.2, 88.3)	67/271 24.7 (19.7, 30.3)	62/279 22.2 (17.2, 27.2)	96/224 42.9 (36.2, 49.5)	158/224 70.5 (64.4, 76.6)
Yellow	7/7650.9 (0.4, 1.9)	65/7658.5 (6.6, 10.7)	111/76514.6 (12.1, 17.3)	635/76583.0 (80.2, 85.6)	205/76526.8 (23.7, 30.1)	166/78421.2 (18.3, 24.1)	250/64238.9 (35.1, 42.8)	451/64270.2 (66.7, 73.8)
Red	6/5111.2 (0.4, 2.5)	56/51111.0 (8.4, 14.0)	117/51122.9 (19.3, 26.8)	482/51194.3 (92.0, 96.2)	181/51135.4 (31.3, 39.7)	104/52619.8 (16.3, 23.2)	200/44245.2 (40.4, 50.1)	307/44269.5 (65.0, 73.9)
Yellow vs green OR (95% CI)	0.82 (0.21, 3.21)	0.88 (0.54, 1.41)	0.87 (0.60, 1.28)	0.92 (0.63, 1.34)	1.11 (0.81, 1.543	0.94 (0.68, 1.31)	0.85 (0.62, 1.16)	0.99 (0.71, 1.38)
Red vs yellow OR (95% CI)	1.29 (0.43, 3.85)	1.32 (0.91, 1.93)	**1.74** **(1.31, 2.33)**	**3.40** **(2.24, 5.18)**	**1.50** **(1.18, 1.91)**	0.92 (0.70, 1.21)	**1.30** **(1.01, 1.66)**	0.96 (0.74, 1.25)
P†	.851	.369	**.003**	**<.001**	**<.001**	.394	.294	.750

Values in bold indicate statistical significance.

CCU, Critical Care Unit; CI, confidence interval; OR, odds ratio.

† All P values are based on the non-parametric test for trend.

**Table 4:** Frequency, post-test probability for outcomes (95% CI) of outcomes across green, yellow and red ‘highest’ traffic light thresholds, odds ratios (95% CI) of yellow vs. green and red vs. yellow traffic lights and non-parametric trend test for worsening traffic light triggers (green to yellow to red).

Outcomes	Maternal death	Eclampsia	Kidney injury	Maternal magnesium sulfate	Maternal CCU admission	Extended perinatal death	Delivery <34 weeks	Delivery <37 weeks
*Post-test probability for outcomes (n,%, 95% CI)*								
Yellow	2/3220.6 (0.1, 2.2)	25/3227.8 (5.1, 11.2)	28/3228.72 (5.9, 12.4)	246/32276.4 (71.4, 80.9)	68/32221.1 (16.8, 26.0)	69/3320.7 (16.3, 25.1)	85/26831.7 (26.0, 37.5)	164/26861.2 (55.2, 67.2)
Red	14/12161.2 (0.6, 1.9)	122/121610.0 (8.4, 11.9)	243/121620.0 (17.8, 22.4)	1093/121689.9 (88.1, 91.5)	385/121631.7 (29.1, 34.4)	258/124720.7 (18.4, 23.0)	460/103644.4 (41.3, 47.5)	750/103672.4 (69.6, 75.2)
Red vs yellow OR (95% CI)	1.86 (0.42, 8.24)	1.32 (0.85, 2.08)	**2.62 (1.73, 3.96)**	**2.75 (2.00, 3.77)**	**1.73 (1.29, 2.32)**	1.00 (0.74, 1.35)	**1.72 (1.29, 2.29)**	**1.66 (1.26, 2.20)**
P†	.373	.139	**<.001**	**<.001**	**<.001**	.415	**<.001**	**<.001**

Values in bold indicate statistical significance.

CCU, Critical Care Unit; CI, confidence interval; OR, odds ratio.

† All P values are based on the non-parametric test for trend.

For those triggering a red light compared to yellow light as their ‘admission’ BP, there was a significant increase in kidney injury, maternal use of magnesium sulfate and maternal CCU admission, but not for maternal death, eclampsia, extended perinatal death or preterm delivery (<34 or <37 weeks), which had a consistently high risk across yellow and red lights. Comparing ‘admission’ yellow and green lights, there was no significant difference in any of the outcomes. For those triggering a red compared to yellow light as their ‘highest’ BP, there was a significant increase in kidney injury, maternal use of magnesium sulfate, CCU admission and preterm delivery (<34 or <37 weeks); but not for maternal death, eclampsia or extended perinatal death.

**Fig. 2:**
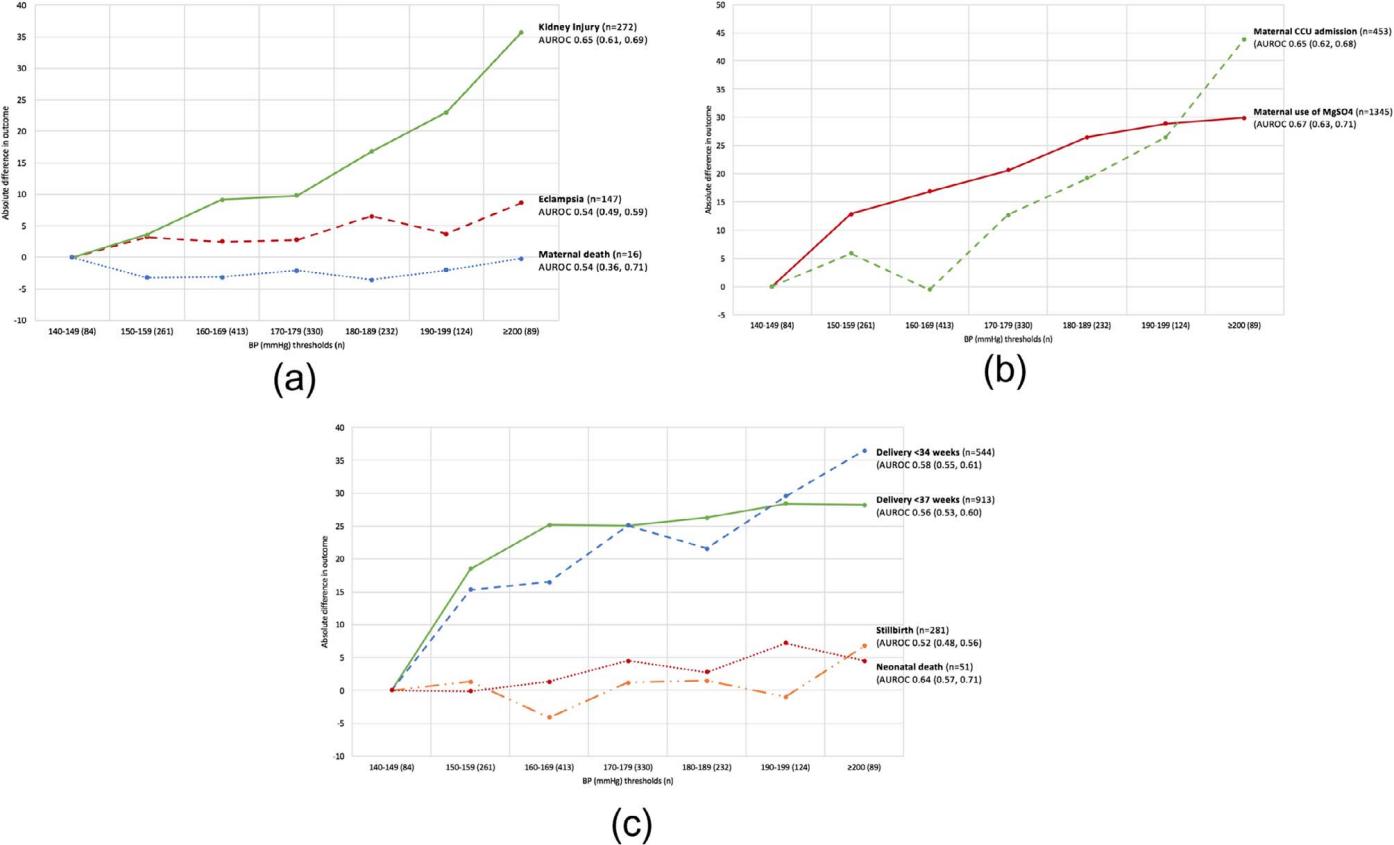
Absolute difference in maternal outcomes (panel A), process outcomes (panel B), perinatal outcomes (panel C) at increasing systolic BP (‘highest’ during admission) from 140 mmHg and the area under the receiver operating characteristic curve (AUROC) values for the performance of highest SBP to predict outcomes, with incidence (%) of outcomes shown above.

Fig. 2 shows the association between ‘highest’ SBP and clinical outcomes, according to AUROC values (95% confidence intervals), and the association between increasing ‘highest’ SBP and absolute differences in outcomes.

Stepwise logistic regression analysis showed that outcomes were consistently poor across the BP range. However, there was no consistent inflection point of either systolic or diastolic BP for ‘admission’ or ‘highest’ BP that demonstrated a change in outcomes; this included at the traffic light thresholds of SBP 140 mmHg, SBP 160 mmHg, DBP 90 mmHg and DBP 110 mmHg. ‘Highest’ SBP ≥210 mmHg was associated with a significantly increased risk of kidney injury, maternal CCU admission, pre-term delivery <34 weeks and stillbirth, but not other outcomes.

## 4 Discussion

### 4.1 Statement of principal findings

The risk of maternal death, eclampsia, and perinatal death was similar across the women who triggered a yellow or red light on the CRADLE VSA. The risk of kidney injury, maternal use of magnesium sulfate, maternal CCU admission and preterm delivery, was greater for those who triggered a red light, compared to a yellow light.

### 4.2 Strengths and weaknesses of the study

Previous literature has focused on the association between pre-diagnosis BP and subsequent development of pre-eclampsia. This prospective observational study of a large multi-centre cohort of pre-eclamptic women assessed the association between CRADLE VSA BP thresholds (at and during admission) and pre-eclampsia complications.

This study ensured the use of accurate BP devices, validated for use in pregnancy including pre-eclampsia, for BP measurement in a pre-eclamptic cohort (rare in previous literature). This is important because the majority of commercially available automated BP devices have not been validated for use in pregnancy including pre-eclampsia and consistently underestimate BP in women with pre-eclampsia [20]. The use of non-validated BP devices for clinical studies involving pre-eclamptic women raises the question of accuracy of the “test” in these studies and possible underestimation of true BP. In this study, the association between accurate BP values and complications of pre-eclampsia was explored.

It was not feasible to collect reliable data on choice and timing of antihypertensive, timing of magnesium sulfate administration and timing of eclampsia and stroke in relation to delivery, due to lack of systematic documentation in this setting. It was not possible to determine timing of stillbirth; often diagnosis was made at admission but could have occurred prior to admission. Therefore, assessing associations with antihypertensive use, temporal trends, and comparisons between antepartum and postpartum eclampsia was not possible. For example, it was possible for eclampsia and peripheral clinic antihypertensive and magnesium sulfate administration to have taken place prior to hospital admission. This may, in part, explain why the BP thresholds were not strongly associated with some outcomes, including eclampsia.

The clinicians were using the BP devices provided by the study as part of routine practice and were not blinded to the BP readings. The decision to use magnesium sulfate and admit to maternal CCU may well have been in response to BP readings. Each study site followed similar departmental guidelines for the management of pre-eclampsia. Guidelines included the recommendation that magnesium sulfate should be administered in women with pre-eclampsia with severe hypertension (systolic BP ≥160 mmHg and/or diastolic BP ≥110 mmHg) or in symptomatic pre-eclampsia without severe hypertension. Those requiring magnesium sulfate may have also been managed in CCU. The association between red traffic light and increasing ‘highest’ SBP and these process measures reflects appropriate response to severe hypertension, but may limit their use as independent outcomes.

At one of the sites (Kimberley Hospital), the proportion of women admitted to CCU was higher than at the other two sites. This can be explained by the criteria at which CCU admission was mandated at that site; Kimberley Hospital had a lower threshold for CCU admission, which tended to care for less severely unwell patients than the other two sites. The relationship between severity of hypertension and CCU admission exists despite this variation between sites.

### 4.3 Strengths and weaknesses in relation to other studies

National and international guideline BP thresholds recommendations are not robustly evidence-based [9], [11], [12], [13], [14], [21]. A recent prospective multicentre study of 2023 pre-eclamptic women demonstrated a relationship between both SBP and DBP and adverse outcome. However specific thresholds of BP were not evaluated and adverse outcomes in this high-income setting were far lower than in our cohort [22]. A similar prospective study of 2081 hypertensive women from five LMICs demonstrated a relationship between SBP and a composite adverse maternal outcome [23]. Although in a more comparable cohort of women and with similar aims to our study, again thresholds of BP were not evaluated.

### 4.4 Meaning of study – Explanations

Women triggering a red traffic light at some point during their admission had a higher risk of kidney injury, preterm delivery and process measure outcomes. These outcomes may have been a consequence of the uncontrolled hypertension and subsequent decisions to intervene (i.e. to deliver the baby) and may not be independent of the severity of hypertension. It was not possible to distinguish between acute kidney injury as a consequence of pre-eclampsia and hypertension as a consequence of chronic renal disease, as baseline creatinine levels were not known. Kidney injury is usually an acute complication in women in low-income countries [24]. This is common to many pregnancy populations where women will not have had a baseline creatinine measured.

A red light did not confer additional risk for maternal death, eclampsia, stillbirth and neonatal death. These findings are consistent with a Haitain pre-eclampsia cohort demonstrating ‘highest’ SBP and DBP during admission were not associated with additional risk of maternal death, eclampsia or antepartum stillbirth [25]. The poor relationship between eclampsia and increasing ‘highest’ SBP mirror findings from a secondary analysis study of 87 women with eclampsia and neuroimaging findings of posterior reversible leuco-encephalopathy syndrome, which showed that more than a third of women had BPs within normal limits (<140/90 mmHg) prior to their eclampsia [26]. A prospective observational study of all eclampsia cases in the UK in 1992 demonstrated that only 38% of in-hospital eclampsia cases were associated with documented proteinuria or hypertension prior to the fit [27]. In our study, risk of eclampsia does not have a close relationship with severity of hypertension. It is possible that eclampsia may occur at moments of acute severe hypertension, which may not always be captured.

In non-pregnant populations, there is a strong association between increasing systolic BP and risk of stroke [28], [29], [30]. The association between stroke risk and severe systolic hypertension is not robust in obstetric populations. In 2005, in 28 women with sustained pre-eclampsia-related strokes, all had a SBP ≥155 mmHg just prior to the stroke [31]. In our data systolic BPs above this threshold were common, yet strokes were rare (despite the four women with strokes also having severe hypertension). This may reflect appropriate and timely management with antihypertensives, magnesium sulfate, CCU admission and delivery of the baby in response to severe hypertension. In lower-resourced settings, the association between severe hypertension and adverse outcomes may be stronger.

### 4.5 Meaning of study – Implications for clinicians/policy

This study aimed to inform whether the BP thresholds incorporated in the CRADLE VSA traffic light early warning system are appropriate as triage tools for healthcare providers caring for pregnant women in low-resource community settings. The study demonstrated that pre-eclamptic women who trigger a yellow or red traffic light are at increased risk of complications of pre-eclampsia, but not for all outcomes. Although the relationship between severity of hypertension and risk of some adverse outcomes, such as eclampsia and stroke, was not strong, these findings should not deter from accurate BP measurement and timely intervention. As discussed above, in the tertiary care setting, treatment paradox and temporal influences may have impacted on the strength of the association.

In a community setting, accurate BP measurement is a critical screening test. In this unselected population, the CRADLE VSA’s yellow light will identify women who are hypertensive (possibly due to pre-eclampsia), at increased risk of a number of pre-eclampsia complications, and who need urgent referral to facility-level care. This should be more urgent when a red light is triggered. The traffic light early warning system enables healthcare providers with limited training to do this without requiring literacy.

### 4.6 Unanswered questions and future research

The traffic light early warning system within the CRADLE VSA device alerts healthcare providers to hypertension and also to shock secondary to obstetric haemorrhage or sepsis. A concurrent study at the same three South African sites evaluated whether thresholds of shock index (the ratio of pulse to SBP) [32] can predict adverse outcomes relating to obstetric haemorrhage and sepsis. Following these two studies, we will assess whether implementation of the CRADLE VSA and a simple training package to healthcare providers caring for pregnant women in low-resource community- and facility-level settings improves outcomes for women (CRADLE 3 Trial), by improving the identification of the three leading causes of maternal death (pre-eclampsia, obstetric haemorrhage and sepsis).
